# pDobz/pDobb protected diaminodiacid as a novel building block for peptide disulfide-bond mimic synthesis†

**DOI:** 10.1039/c8ra09761e

**Published:** 2019-02-12

**Authors:** Chao Liu, Yan Zou, Honggang Hu, Yunyun Jiang, Luping Qin

**Affiliations:** College of Pharmacy, Second Military Medical University Shanghai China; Department of Pharmacy No. 101 Hospital of PLA Wuxi China Shygm98@163.com; College of Pharmaceutical Sciences, Zhejiang Chinese Medical University Hangzhou China qinsmmu@126.com

## Abstract

The diaminodiacid strategy has been widely studied in the chemical synthesis of peptide disulfide bond mimics. Diaminodiacid building blocks, which are key intermediates, are currently under the spotlight. However, one technical bottleneck inherent in existing building blocks is the contamination problem caused by the heavy metal reagents during the deprotection process, which makes the peptides less suitable for pharmaceutical use. Herein, we describe the successful development of a *p*-dihydroxyborylbenzyloxycarbonyl pinacol ester (pDobz)- and *p*-dihydroxyborylbenzyl pinacol ester (pDobb)-based novel diaminodiacid building block that can be easily deprotected *via* mild treatment with amine oxide. Its efficiency and practicability were also confirmed by the total synthesis of contryphan-Vn disulfide bond mimic. The results suggested that this novel diaminodiacid building block has satisfactory Fmoc SPPS compatibility, yet only required a facile, rapid, and metal-free deprotection process. We believe this novel diaminodiacid building block could promote further development of the diaminodiacid strategy.

## Abstract 2

The total synthesis of a contryphan-Vn disulfide bond mimic utilizing a novel pDobz/pDobb based diaminodiacid building block *via* the diaminodiacid strategy.

## Graphical abstract

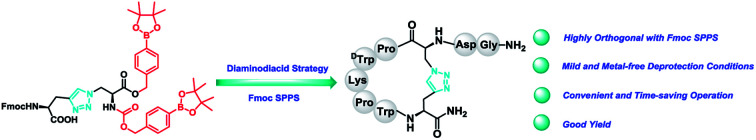

## Introduction

Disulfide bonds play essential and indispensable roles in many peptides and proteins. They help proteins maintain their metabolic stability, biological activity, and target selectivity.^1–5^ Studies have shown about one-fourth of the peptidic molecules in the protein data bank (PDB) contain at least one disulfide bridge.^6^ Many of them are under clinical or pre-clinical study for their potential therapeutic roles in pain disorders, cancer, coagulation disorders, and other conditions.^7–10^ However, disulfide bonds are unstable *in vivo* because of reduction, disulfide isomerases, and enzymatic cleavage; the degradation then leads to structural distortion and activity loss.^11,12^ To overcome these roadblocks, a number of approaches to synthetic disulfide surrogates with improved redox stability and conformation rigidity have been developed. These include thioether, olefin, diselenide, and triazole bridges.^13–18^

**Fig. 1:**
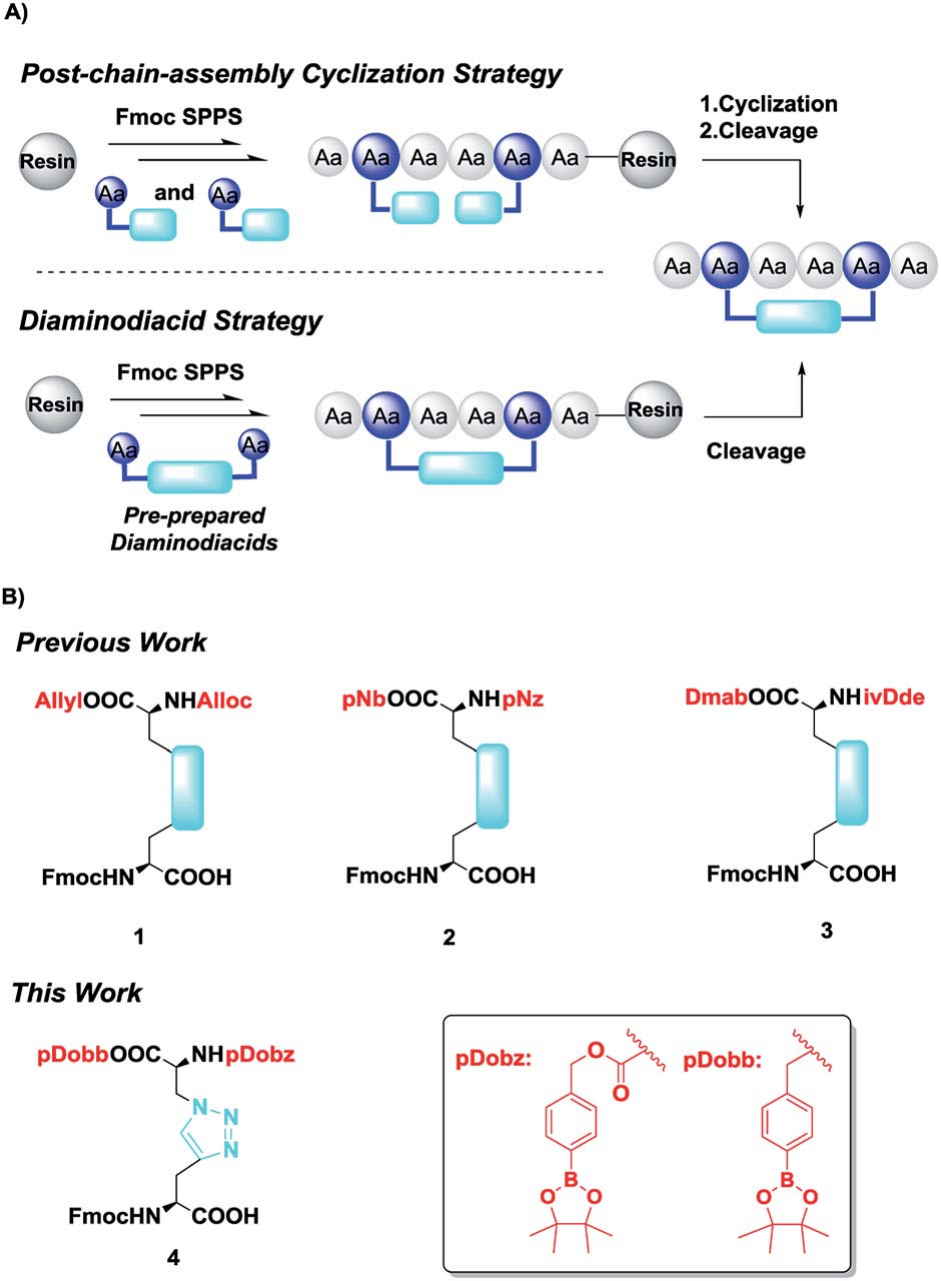
(A) General strategy for post-chain-assembly cyclization and diaminodiacid-based SPPS; (B) previously reported and novel diaminodiacid building blocks.

The diaminodiacid strategy is one alternative to the post-chain-strategy as a means of inserting these disulfide replacements into the peptide backbone. This is because few types of disulfide surrogates can be realized *via* the post-chain-strategy.^19^ As shown in Fig. 1A, pre-prepared diaminodiacid building blocks can be readily installed through an amide coupling reaction rather than generation nonpeptidic bridge on solid support, which requires harsh reaction conditions and often has unsatisfactory yield.^19–21^ More importantly, when multiple disulfide surrogates need to be introduced, the diaminodiacid strategy can overcome the regioselectivity misfolding problem associated with post-chain-assembly cyclization.^19,22^

**Fig. 2:**
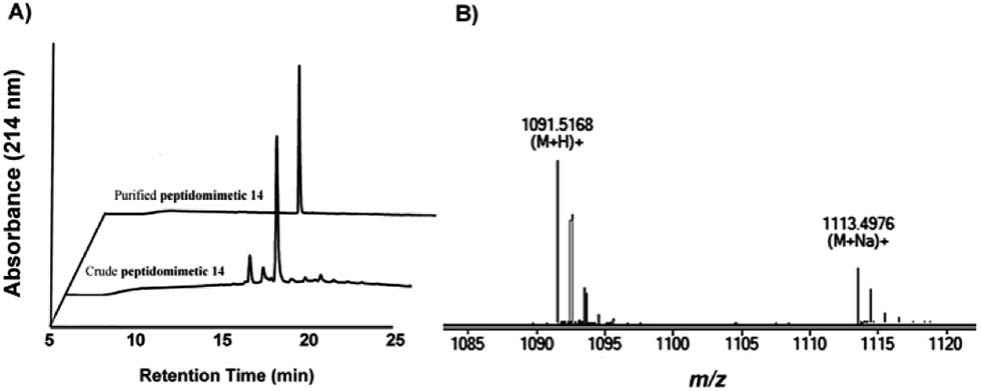
(A) HPLC traces of crude and purified 14. (B) HRMS spectrum of 14, calcd for C_52_H_66_N_16_O_11_ 1090.5097, found [M + H]^+^ 1091.5168, and found [M + Na]^+^ 1113.4976.

**Fig. 3:**
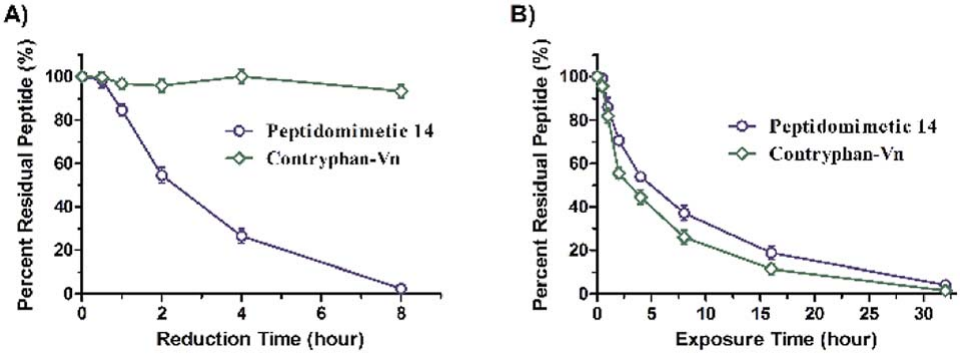
Reduction (A) and protease (B) stability of 14*vs.* contryphan-Vn under DTT or α-chymotrypsin treatment. Data points are displayed as the mean value SEM of duplicate independent experiments. The percent residual peptide was monitored *via* HPLC.

In previous studies, diaminodiacid building blocks were frequently orthogonally capped by allyloxycarbonyl (Alloc)/Allyl or *p*-nitrobenzyloxycarbonyl (pNZ)/*p*-nitrobenzyl (pNB) protective groups (Fig. 1B, 1 and 2). These two pairs of protecting groups can be removed utilizing Pd(PPh_3_)_4_/PhSiH^23^ and SnCl_2_/HCl,^24,25^ respectively, on solid support. However, the use of heavy metal reagents may cause detrimental contamination during peptide and protein preparation, especially for pharmaceutical use.^22,26^ Besides, the use of protective inert atmosphere and the time-consuming process hinder the convenient operation demanded by SPPS. To overcome this problem, Xu *et al.*^26^ described the utility of 4-(*N*-[1-(4,4-dimethyl-2,6-dioxocyclohexylidene)-3-methylbutyl]-amino)benzyl (Dmab)/1-(4,4-dimethyl-2,6-dioxocylcohex-1-ylidene)-3-meth-ylbutyl (ivDde) as diaminodiacid protection groups (Fig. 1B, 3) that can be removed by treatment with 2% hydrazinolysis in DMF solution. Nevertheless, some of degradation of Dmab/ivDde caused by repeated deprotection of the Fmoc group using 20% piperidine in DMF solution during SPPS can lead to undesirable byproducts, which may hinder the comprehensive application of this kind of diaminodiacid building block. Although this strategy can be optimized to some extent by shortening the duration of each piperidine treatment or by using 2-methylpiperidine as a deprotection reagent, the discovery and development of a novel diaminodiacid building block, which is fully orthogonal with Fmoc SPPS and can be effectively deprotected under metal-free conditions, still remains a field of intense research. We took inspiration from a series of arylboronate ester-based amino acid building blocks recently developed by our team.^27^

The pDobz/pDobb protective groups were found to be stable with acid and base,^27,28^ fully compatible with Fmoc SPPS and can be quickly and effectively removed in 30 minutes *via* treatment with amine oxide and the following low concentration of acid.^27^

By taking these advantages into account, we here demonstrate the successful development of a pDobz/pDobb-protected diaminodiacid building block bearing a 1,4-disubstituted 1,2,3-triazole bridge (Fig. 1B, 4) which was extensively applied in peptide chemistry because of its extraordinary thermal and metabolic stability.^17,29,30^ Besides, we confirmed the efficiency and practicality of this novel diaminodiacid building block by preparing disulfide bond mimetic of the model peptide contryphan-Vn, a disulfide-constrained nonapeptide, which is a modulator of Ca^2+^-dependent K^+^ channels.^31,32^ This work makes it possible to provide a better key intermediate for use with the diaminodiacid strategy.

## Results and discussion

**Scheme 1:**
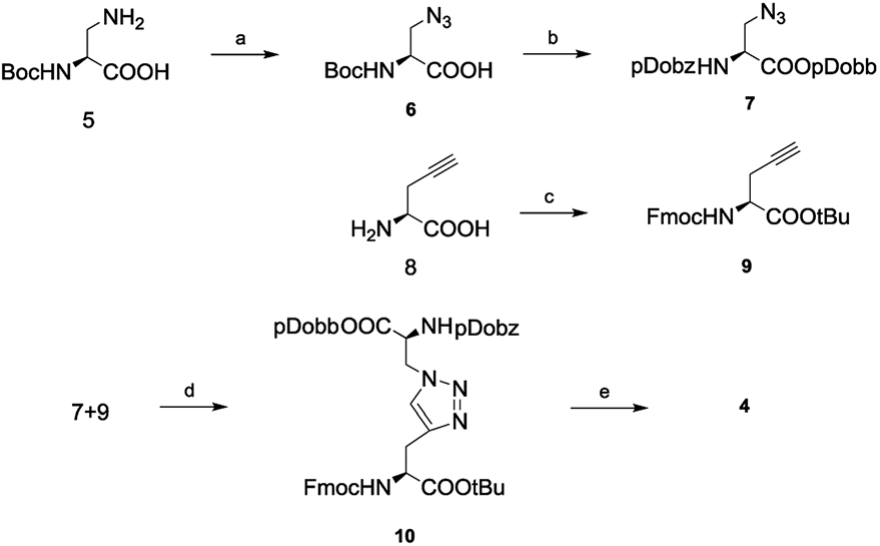
Route of synthesis of novel diaminodiacid building block; reagents and conditions: (a) TfN_3_, CuSO_4_·5H_2_O, K_2_CO_3_, water/MeOH/DCM, rt, 12 h, 78%; (b) (i) 4 M HCl/1,4-dioxane, rt, 2 h; (ii) pDob-N_3_, Et_3_N, DMF, rt, 12 h; (iii) pDobb-OH, DCC, DMAP, DCM, rt, 12 h, 65% in three steps; (c) (i) FmocOSu, Na_2_CO_3_, water/1,4-dioxane; (ii) *t*BuOH, DCC, DMAP, DCM, rt, 12 h, 71% in two steps; (d) CuI, DIPEA, THF, rt, 12 h, 86%; (e) TFA/DCM (1 : 1, v/v), rt, 2 h, 96%.

At the beginning of our study, the pdob/pdobb-protected 1,4-disubstituted 1,2,3-triazole bridge-based diaminodiacid (4) was synthesized (Scheme 1). Initially, the 3-amino-*N*-Boc-l-alanine acid (Boc-Dap-OH, 5) was converted to 3-azido-*N*-Boc-l-alanine acid (Boc-N_3_-OH, 6) through a Cu(ii)-catalyzed diazo-transfer reaction intrigued with freshly prepared trifluoromethanesulfonyl azide (TfN_3_) in a good yield of 78%. The *tert*-butoxycarbonyl (Boc) protection group was then readily removed by the treatment with hydrogen chloride in ethyl acetate solution. The resulting hydrochloride was reacted with 4-(4,4,5,5-tetramethyl-1,3,2-dioxaborolan-2-yl)benzyl carbonazidate (pDobz-N_3_) in the presence of Et_3_N as described in our previous report.^27^ Then, a pDobb protective group was readily installed on the carboxyl through an esterification reaction with 4-(4,4,5,5-tetramethyl-1,3,2-dioxaborolan-2-yl)phenyl methanol (pDobb-OH), *N*,*N*-dicyclohexylcarbodiimide (DCC), and catalytic 4-dimethylaminopyridine (DMAP) to provide the fully-protected pDobz-N_3_-pDobb (7) at a yield of 65%. Subsequently, the capping of the Fmoc group on 2- propargyl-l-glycine (H-Pra-OH, 8) was successfully realized using FmocOSu. The following *tert*-butyl (*t*Bu) protection generated the Fmoc-Pra-OtBu (9) with a 71% yield in two steps. Then, 7 and 9 were azide–alkyne cycloaddition (CuAAC) by CuI and *N*,*N*-diisopropylethylamine (DIPEA) to produce the protected diaminodiacid (10). Finally, *t*Bu protection group could be easily cleaved with trifluoroacetic acid in dichloromethane solution to provide the target diaminodiacid (4) at a high yield of 96%.

**Scheme 2:**
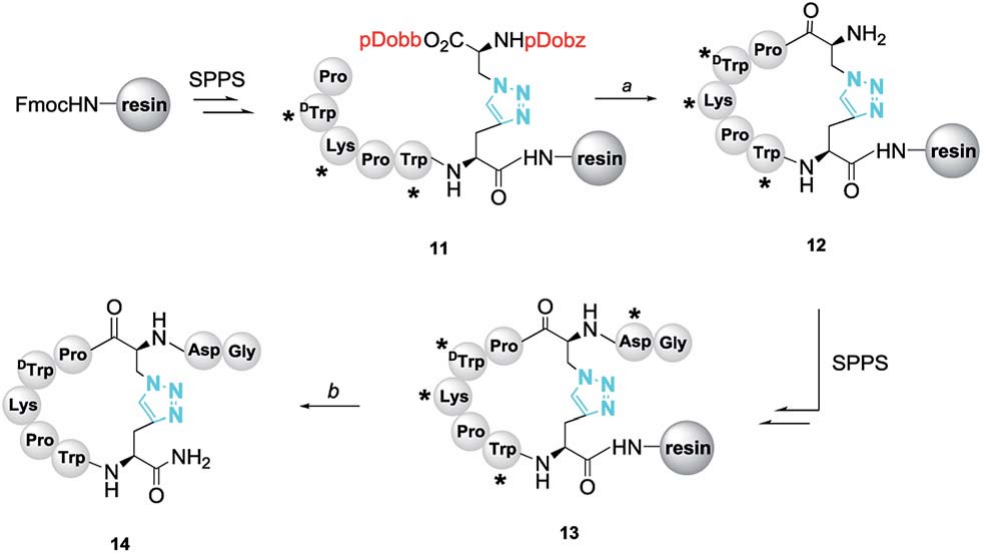
Route of synthesis of contryphan-Vn disulfide bond mimic; reagents and conditions: (a) (i) *N*-methyl-*N*-phenylaniline *N*-oxide, DCM, rt, 15 min; (ii) TFA/*m*-cresol/DCM (5 : 20 : 75), v/v/v, rt, 15 min; (iii) 20% piperidine/DMF, rt, 10 min; (iv) PyAOP, HOAt, NMM, NMP, rt, 12 h; (b) TFA/EDT/TIPs/water (95 : 2 : 2 : 1, v/v/v/v), rt, 2 h. The resin-bound peptides were protected on side chains at asterisk sites. The following protecting groups for amino acid side chains were used: *tert*-butyl (*t*Bu; for Asp) and *tert*-butyloxycarbonyl (Boc; for Lys and Trp).

The synthesis of the peptidomimetics 14 was started from the Rink amide AM resin. As shown in Scheme 2, normal amino acids and 4 were introduced into the peptide backbone on resin (loading = 0.33 mmol g^−1^) using *O*-(6-chloro-1-hydrocibenzotriazol-1-yl)-1,1,3,3-tetramethyluronium hexaflu-orophosphate (HCTU) as the coupling reagent to provide cyclization precursor 11. Then, the pDobz/pDobb protection groups were readily removed by successive treatment of *N*-methyl-*N*-phenylaniline oxide in dichloromethane solution and trifluoroacetic acid/*m*-cresol in dichloromethane solution according to our previous report. After the cleavage of the Fmoc group, intramolecular macrocyclization was successfully accomplished with (7-azabenzotriazol-1-yloxy)tripyrrolidino- phosphonium hexafluorophosphate (PyAOP), 1-hydroxy-7-azabenzotriazole (HOAt), and 4-methylmorpholine (NMM) in *N*-methyl pyrrolidone (NMP) solution to obtain the cyclic peptide 12. The on-resin full-length contryphan-Vn derivative (13) was produced after assembling the remaining aspartic acid and glycine. Finally, acidic cleavage and concomitant global deprotection yielded the crude target peptidomimetic 14.

Further analysis and purification were carried out through reverse-phase high-performance liquid chromatography. As shown in Fig. 2, crude product contained only a single major component that can be easily purified in 98.6% purity, and the resulting total yield was 36% (according to initial resin load). Then, the molecular weight was confirmed using high-resolution mass spectrometry (HRMS) and found to be identical to the theoretical molecular mass. Hence, these results suggested that the novel diaminodiacid building block was highly orthogonal with Fmoc SPPS and fairly efficient for synthesizing peptidomimetic components containing disulfide bond surrogates.

It is important to emphasize that the installation of triazole surrogates in contryphan-Vn improved its stability.^19^ To this end, we synthesized the native contryphan-Vn through Fmoc SPPS and dimethylsulfoxide-mediated solution-phase oxidation cyclization strategy (Scheme S1†). Subsequently, we carried out the reduction stability experiment using dithiothreitol (DTT) as reducing agent.^33^ Indeed, contryphan-Vn was completely reduced in eight hours in aqueous DTT solution, while peptidomimetics 14 remained intact after eight hours' treatment (Fig. 3A). Furthermore, the protease stability experiment was investigated. α-Chymotrypsin is a protease that preferentially cleaves peptide amide bonds where the side-chain of the amino acid *N*-terminal to the scissile amide bond is a large, hydrophobic amino acid such as Trp, Phe and Leu, and is used for peptide protease stability studies.^34^ Contryphan-Vn and peptidomimetics 14 were subjected to the α-chymotrypsin mediated degradation test and monitored by HPLC. It was found that after 32 hours' protease exposure, both of them were degraded completely. No significant differences of protease stability were observed between peptidomimetics 14 and native model peptides (Fig. 3B). These results indicated that the introducing of triazole replacement could significantly improve the reduction stability of peptide rather than the protease stability.

## Conclusions

In summary, to our knowledge, this is the first introduction of arylboronate ester protective groups to diaminodiacid building block construction, and the triazole bridge-based peptidomimetic of contryphan-Vn was also developed and studied for the first time. More importantly, the low environmental cost, facile handling property, and the resulting high purity and good yield of the final product suggest that this novel building block is a better key intermediate for the diaminodiacid strategy, so it may promote further development of disulfide bond mimetic studies in the future.

## Experimental section

### General information

All reagents were purchased from Acros, Sigma-Aldrich, Alfa Aesar and Adamas. Amino acids were commercial available from GL Biochem Shanghai Co. Ltd. All solvents used were bought from Sinopharm Chemical Reagent Co. Ltd. Dichloromethane (DCM) and *N*,*N*-dimethylformamide (DMF) were distilled over calcium hydride (CaH_2_) under argon atmosphere and stored in flask containing 4 Å molecular sieves. All reactions vessels were oven-dried before use. Reactions were monitored by thin-layer chromatography (TLC) and visualized by UV analyzer (254 nm), ninhydrin and/or phosphomolybdic acid. Peptides were analyzed and purified by reverse phase HPLC. A C18 analytic column (Shimazu Shim-pack VP-ODS, 4.6 × 250 mm, 5 μm particle size, flow rate 1 mL min^−1^) was used for analytical RP-HPLC, and a C18 column (Shimazu Shim-pack PRC-ODS, 50 × 250 mm, 15 μm particle size, flow rate 10 mL min^−1^) was used for preparative RP-HPLC. The solvents systems were buffer A (0.1% TFA in CH_3_CN) and buffer B (0.1% TFA in water). Data were recorded and analyzed using the software system LC Solution. High resolution mass spectra (HR-MS) were measured on a Waters Xevo G2 QTOF mass spectrometer. ^1^H- and ^13^C-NMR spectrum was recorded on a Bruker Avance 300 MHz instrument. Chemical shifts (*δ*) were reported relative to TMS (0 ppm) for ^1^H- and ^13^C-NMR spectra. The coupling constants (*J*) were displayed in hertz (Hz) and the splitting patterns were defined as follows: singlet (s); broad singlet (s, br); doublet (d); doublet of doublet (dd); triplet (t); quartet (q); multiplet (m).

### (*S*)-3-Azido-2-((*tert*-butoxycarbonyl)amino)propanoic acid (6)

To a solution of NaN_3_ (4.8 g, 74.0 mmol) in DCM/water (2 : 1, v/v, 40.0 mL) was added Tf_2_O (2.5 mL, 14.8 mmol) dropwise over 15 minutes at 0 °C. The reaction mixture was stirred vigorously at 0 °C for 2 hours. The organic phase was separated, washed with saturated Na_2_CO_3_ (3 × 100.0 mL) and used without further purification. To a mixture of commercially available Boc-Dap-OH (1.5 g, 7.4 mmol, 5), K_2_CO_3_ (2.0 g, 14.8 mmol) and catalytic amount CuSO_4_·5H_2_O in water/MeOH (2 : 1, v/v, 70.0 mL) was added freshly prepared TfN_3_ solution dropwise at 0 °C, then more MeOH was added to homogeneity. The mixture was allowed to warm to room temperature and stirred overnight. The DCM and MeOH were removed under vacuum and the aqueous solution was acidified by 1 M HCl to pH = 2. After dilution with EtOAc (100.0 mL), the organic layer was separated, washed with brine (3 × 100.0 mL), dried over Na_2_SO_4_, concentrated and purified by column chromatography (100 : 1–10 : 1, DCM/MeOH) to give 6 as a colorless oil (1.3 g, 78%). ^1^H-NMR (300 MHz, *d*-DMSO): *δ* 7.28 (d, *J* = 9 Hz, 1H), 4.15–4.08 (m, 1H), 3.55–3.51 (m, 2H), 1.37 (s, 9H). ^13^C-NMR (300 MHz, *d*-DMSO): *δ* 171.73, 155.78, 78.93, 53.88, 51.29, 28.60. HR-MS *m*/*z* calcd for C_8_H_14_N_4_O_4_ 230.1015; found [M − H]^−^ 229.1149.

### 4-(4,4,5,5-Tetramethyl-1,3,2-dioxaborolan-2-yl)benzyl(*S*)-3-azido-2-((((4-(4,4,5,5-tetramethyl-1,3,2-dioxaborolan-2-yl)benzyl)oxy)carbonyl)amino)propanoate (7)

6 (1.3 g, 5.6 mmol) was dissolved in 4 M HCl/1,4-dioxane (20.0 mL) at 0 °C. The reaction mixture was stirred for 2 hours at room temperature. Then the resulting mixture was filtrated and the solid was washed with Et_2_O twice. After dried over vacuum, the solid was used directly in the next step. To a solution of 4-(4,4,5,5-tetramethyl-1,3,2-dioxaborolan-2-yl)benzyl carbonazidate (pDobz-N_3_, 1.7 g, 5.6 mmol) and prepared hydrochloride in dry DMF (50.0 mL) was added TEA (2.3 mL, 16.8 mmol). The reaction mixture was stirred for 24 hours at room temperature. The mixture was evaporated to dryness and diluted with EtOAc (100.0 mL) and water (100.0 mL). The organic layer was successively separated, washed with saturated 1 M HCl (3 × 100.0 mL), brine (3 × 100.0 mL), dried over Na_2_SO_4_, filtered and concentrated which was used directly in the next step. To a solution of crude pDobz protected amino acid, (4-(4,4,5,5-tetramethyl-1,3,2-dioxaborolan-2-yl)phenyl)methanol (pDobb-OH, 1.3 g, 5.6 mmol) and DMAP (68 mg, 0.56 mmol) in DCM (100.0 mL) was added DCC (1.2 g, 5.6 mmol) at 0 °C. The mixture was stirred for 1 hour at 0 °C and 4 hours at room temperature. Then, the reaction was filtrated and the filtrates were washed successively with 1 M HCl (3 × 100.0 mL), saturated NaHCO_3_ (3 × 100.0 mL) and brine (3 × 100.0 mL). The organic phase was dried over Na_2_SO_4_, filtered, concentrated and purified by column chromatography (50 : 1–10 : 1, petro ether/EtOAc) to give 7 as a colorless oil (2.2 g, 65%, 3 steps). ^1^H-NMR (300 MHz, CDCl_3_): *δ* 7.82–7.78 (m, 4H), 7.35–7.33 (m, 4H), 5.69 (d, *J* = 9 Hz, 1H), 5.29 (s, 1H), 5.22 (s, 2H), 5.14 (s, 2H), 4.58–4.56 (m, 1H), 1.34 (s, 24H). ^13^C-NMR (300 MHz, CDCl_3_): *δ* 169.26, 155.81, 138.85, 137.65, 135.14, 135.04, 127.50, 127.12, 84.04, 83.98, 67.86, 67.31, 53.99, 53.44, 52.53, 24.83. HR-MS *m*/*z* calcd for C_30_H_40_B_2_N_4_O_8_ 606.3032; found [M + H]^+^ 607.3137.

### *tert*-Butyl–(*S*)-2-((((9*H*-fluoren-9-yl)methoxy)carbonyl)amino)pent-4-ynoate (9)

To a solution of commercial available H-Pra-OH (2.5 g, 22.1 mmol, 8) in water/1,4-dioxane (1 : 1, v/v, 100.0 mL) was added NaHCO_3_ (3.9 g, 44.2 mmol) and FmocOSu (14.9 g, 44.2 mmol) successively at 0 °C. Then the mixture was stirred overnight at room temperature. After the 1,4-dioxane was removed under vacuum, the aqueous phase was acidified with 1 M HCl to pH = 2 and extracted with EtOAc (2 × 100.0 mL). The organic layer was washed with brine (3 × 100.0 mL), dried over Na_2_SO_4_ and concentrated to provide crude Fmoc-Pra-OH which was used directly without further purification. To a solution of the crude Fmoc-Pra-OH, *tert*-butanol (2.0 mL, 22.1 mmol) and DMAP (0.3 g, 2.2 mmol) in DCM (100.0 mL) was added DCC (4.5 g, 22.1 mmol) at 0 °C. The mixture was stirred for 1 hour at 0 °C and 4 hours at room temperature. Then, the reaction was filtrated and the filtrates were washed successively with 1 M HCl (3 × 100.0 mL), saturated NaHCO_3_ (3 × 100.0 mL) and brine (3 × 100.0 mL). The organic phase was dried over Na_2_SO_4_, filtered, concentrated and purified by column chromatography (50 : 1–10 : 1, petro ether/EtOAc) to give 9 as a light yellow powder (6.1 g, 71%, 2 steps). ^1^H-NMR (300 MHz, CDCl_3_): *δ* 7.76 (d, *J* = 9 Hz, 2H), 7.61 (d, *J* = 9 Hz, 2H), 7.43–7.38 (m, 2H), 7.34–7.29 (m, 2H), 5.68 (d, *J* = 6 Hz, 1H), 4.45–4.37 (m, 3H), 4.27–4.22 (m, 1H), 2.77 (s, 2H), 1.50 (s, 9H). ^13^C-NMR (300 MHz, CDCl_3_): *δ* 169.32, 155.61, 143.87, 143.79, 141.30, 127.73, 127.09, 125.17, 120.00, 82.89, 78.56, 71.55, 67.20, 52.64, 47.13, 27.98, 23.00. HR-MS *m*/*z* calcd for C_24_H_25_NO_4_ 391.1784; found [M + H]^+^ 392.1682.

### *tert*-Butyl-2-((((9*H*-fluoren-9-yl)methoxy)carbonyl)amino)-3-(1-(3-oxo-3-((4-(4,4,5,5-tetramethyl-1,3,2-dioxaborolan-2-yl)benzyl)oxy)-2-((((4-(4,4,5,5-tetramethyl-1,3,2-dioxaborolan-2-yl)benzyl)oxy)carbonyl)amino)propyl)-1*H*-1,2,3-triazol-4-yl)propanoate (10)

To a solution of 7 (1.2 g, 2.0 mmol), 9 (794 mg, 2.0 mmol) and CuI (570 mg, 3.0 mmol) in dry THF (20.0 mL) was added DIPEA (3.5 mL, 20.0 mmol) under Ar atmosphere. Protected from light, the reaction mixture was stirred for 14 hours at room temperature. The resulting mixture was diluted with EtOAc (100.0 mL) and water (100.0 mL). The organic phase was filtered, washed with 1 M HCl (3 × 100.0 mL), brine (3 × 100.0 mL), dried over Na_2_SO_4_, filtered, concentrated and purified by column chromatography (10 : 1–1 : 1, petro ether/EtOAc) to give 10 as a white powder (1.7 g, 86%). ^1^H-NMR (300 MHz, CDCl_3_): *δ* 7.82–7.72 (m, 6H), 7.58 (d, *J* = 6 Hz, 2H), 7.40–7.29 (m, 8H), 7.14–7.08 (m, 1H), 5.78–5.66 (m, 2H), 5.19 (s, 2H), 5.13–5.07 (m, 2H), 4.81–4.75 (m, 3H), 4.67–4.54 (m, 1H), 4.35 (d, *J* = 6 Hz, 2H), 4.22–4.15 (m, 1H), 3.22–3.10 (m, 2H), 1.44–1.41 (m, 9H), 1.33 (s, 24H). ^13^C-NMR (300 MHz, CDCl_3_): *δ* 179.37, 168.45, 155.70, 143.85, 141.27, 137.51, 135.14, 135.02, 127.84, 127.68, 127.10, 123.28, 119.93, 83.96, 83.86, 82.55, 68.11, 67.95, 67.17, 67.04, 54.22, 50.74, 48.45, 47.12, 44.40, 27.95, 24.86. HR-MS *m*/*z* calcd for C_54_H_65_B_2_N_5_O_12_ 997.4816; found [M + H]^+^ 998.4977.

### 2-((((9*H*-Fluoren-9-yl)methoxy)carbonyl)amino)-3-(1-(3-oxo-3-((4-(4,4,5,5-tetramethyl-1,3,2-dioxaborolan-2-yl)benzyl)oxy)-2-((((4-(4,4,5,5-tetramethyl-1,3,2-dioxaborolan-2-yl)benzyl)oxy)carbonyl) amino)propyl)-1*H*-1,2,3-triazol-4-yl)propanoic acid (4)

10 (1.7 g, 1.7 mmol) was dissolved in TFA/DCM (1 : 1, v/v, 30.0 mL) and stirred for 2 hours at room temperature. The reaction mixture was concentrated *in vacuo* to yield 4 as a white powder (1.5 g, 96.0%) which was used directly in SPPS without further purification. ^1^H-NMR (300 MHz, CDCl_3_): *δ* 7.81–7.71 (m, 6H), 7.55 (s, 2H), 7.38–7.29 (m, 8H), 7.08 (s, 1H), 6.00–5.75 (m, 2H), 5.23–5.06 (m, 3H), 4.73 (s, 3H), 4.34 (s, 2H), 4.18 (s, 1H), 3.28 (s, 2H), 1.33 (s, 24H). ^13^C-NMR (300 MHz, CDCl_3_): *δ* 172.63, 168.25, 156.15, 143.88, 143.72, 141.27, 137.51, 135.14, 135.02, 127.98, 127.88, 127.74, 127.15, 125.17, 119.94, 83.89, 75.50, 68.16, 67.39, 54.00, 53.15, 51.11, 47.02, 24.82. HR-MS *m*/*z* calcd for C_50_H_57_B_2_N_5_O_12_ 941.4190; found [M − H]^−^ 940.4025.

### General procedures for the Fmoc solid phase peptide synthesis

The amino acid residues were attached to the resin with a single coupling procedure. All peptides were synthesized with a scale of 0.10 mmol.

(a) Standard pre-activation of resin protocol: the resin was swollen in DCM/DMF mixture solvent for 10 minutes.

(b) Standard Fmoc-deprotection protocol: after treatment with 20% piperidine in DMF solution (15 minutes twice) the resin was washed with DMF (×5), DCM (×5), and DMF (×5).

(c) Standard coupling of natural amino acids protocol: after pre-activation of 4.0 equivalents of Fmoc-protected amino acid in DMF for 5 minutes using 3.8 equivalents of HCTU and 8.0 equivalents of DIPEA, the solution was added to the resin. After 30 minutes, the resin was washed with DMF (×5), DCM (×5), and DMF (×5). The coupling reaction was monitored with the ninhydrin test.

(d) Standard coupling of diaminodiacids protocol: after pre-activation of 1.5 equivalents of Fmoc-protected diaminodiacid in DMF for 1 minute using 2.0 equivalents of HATU and 8.0 equivalents of DIPEA, the solution was added to the resin. After 2 hours, the resin was washed with DMF (×5), DCM (×5), and DMF (×5). The coupling reaction was monitored with the ninhydrin test.

(e) Standard deprotection of pDobz/pDobb protocol: after treatment with 5 equivalents of *N*-methyl-*N*-phenylaniline oxide in DCM solution for 15 minutes, the resin was washed with DMF (×5), DCM (×5), and DMF (×5). Then a solution of TFA/*m*-cresol/DCM (5 : 20 : 75, v/v/v) was added to the resin. After 15 minutes, the resin was washed with DMF (×5), DCM (×5), and DMF (×5).

(f) Standard cyclization protocol: after removal of pDobz/pDobb and *N*-terminus Fmoc successively, a solution of PyAOP (5.0 equivalents), HOAt (5.0 equivalents) and NMM (10.0 equivalents) in *N*-methyl-2-pyrrolidone solution was added to the resin. After overnight reaction, the resin was washed with DMF (×5), DCM (×5), and DMF (×5).

(g) Standard cleavage protocol: the cleavage cocktail (TFA : EDT : TIPS : water = 95 : 2 : 2 : 1, v/v/v/v) was added to the resin. After stirring for 2 hours, the cleavage cocktail was collected. The solution was bubbled with argon for concentration and the chilled diethyl ether was added to precipitate the crude peptides. The peptide suspensions were centrifuged for 3 minutes at 3000 rpm and then the clear solution was decanted. The step of precipitation, centrifugation and decantation operations was repeated three times. The resulting white residues were dissolved in CH_3_CN/water, analyzed and purified by RP-HPLC.

(h) Standard oxidative folding protocol: peptide in the reduced form was dissolved in the oxidation buffer (0.5 mg mL^−1^ peptide in 6.0 M guanidine hydrochloride and 100.0 mM sodium dihydrogen phosphate PBS buffer, pH = 7.4, with 10.0% DMSO). This mixture was allowed to stir for 24 h at room temperature. Then it was analyzed and purified by RP-HPLC.

### [Ala_1_(&^1^),Ala_7_(&^2^)]contryphan-Vn[1,9][(&^1^-1,4-[1,2,3]-triazolyl-&^2^)] (14)

Compound 14 was obtained as a lyophilized white powder (39.5 mg, 36% yield according to initial resin load, 99.3% purity). ^1^H-NMR (300 MHz, *d*-DMSO): *δ* 10.85–10.83 (m, 2H), 8.84–8.57 (m, 1H), 8.10–8.01 (m, 4H), 7.86–7.69 (m, 3H), 7.79–7.69 (d, *J* = 9 Hz, 2H), 7.33–7.31 (d, 3H, *J* = 6 Hz), 7.20–7.13 (m, 4H), 7.06–7.04 (m, 2H), 6.99–6.92 (m, 2H), 4.89–4.85 (m, 1H), 4.52–4.42 (m, 3H), 4.31–4.29 (m, 2H), 4.19–4.14 (m, 2H), 3.56–3.52 (m, 5H), 3.42 (s, 2H), 3.21–3.17 (m, 2H), 3.05–3.01 (m, 2H), 2.77–2.75 (m, 2H), 2.02–1.91 (m, 2H), 1.74–1.70 (m, 4H), 1.68–1.64 (m, 2H), 1.58–1.46 (m, 6H), 1.31–1.23 (m, 6H). ^13^C-NMR (300 MHz, *d*-DMSO): *δ* 172.60, 172.41, 172.35, 172.24, 171.63, 171.52, 170.35, 158.99, 158.78, 158.57, 158.35, 130.11, 127.84, 127.71, 124.17, 124.09, 121.32, 120.35, 118.37, 114.42, 111.03, 110.46, 110.40, 110.27, 62.18, 62.07, 60.60, 60.28, 60.14, 59.93, 55.53, 54.45, 53.32, 50.44, 49.96, 37.87, 37.62, 30.91, 29.49, 29.18, 27.24, 27.16, 24.72, 24.37, 23.94, 22.29, 22.22, 14.40. HR-MS *m*/*z* calcd for C_50_H_57_B_2_N_5_O_12_ 1090.5097, found [M + H]^+^ 1091.5168, and found [M + Na]^+^ 1113.4976.

### Contryphan-Vn[1,9]

Contryphan-Vn was obtained as a white lyophilized powder (46.8 mg, 43% yield according to initial resin load, 97.2% purity). ^1^H-NMR (300 MHz, *d*-DMSO): *δ* 10.86–10.83 (m, 2H), 8.85–8.68 (m, 1H), 8.07–8.04 (m, 3H), 7.82–7.77 (m, 4H), 7.58–7.54 (m, 2H), 7.33–7.31 (m, 2H), 7.22 (s, 2H), 7.17–7.13 (m, 2H), 7.07–7.04 (m, 2H), 6.99–6.95 (m, 2H), 4.68–4.52 (m, 2H), 4.49–4.43 (m, 3H), 4.32–4.22 (m, 3H), 3.57 (s, 3H), 3.43–3.38 (m, 2H), 3.21–3.12 (m, 2H), 2.96–2.92 (m, 1H), 2.79–2.66 (m, 6H), 2.58–2.52 (m, 2H), 2.20–2.17 (m, 1H), 2.00–1.91 (m, 2H), 1.76–1.74 (m, 4H), 1.64–1.47 (m, 6H), 1.29–1.23 (m, 5H). ^13^C-NMR (300 MHz, *d*-DMSO): *δ* 172.61, 171.84, 171.59, 171.47, 171.22, 170.76, 169.75, 166.42, 166.14, 159.07, 158.42, 136.50, 136.46, 130.11, 127.79, 121.30, 118.74, 118.64, 116.26, 114.29, 111.79, 111.71, 110.48, 110.35, 60.36, 60.16, 55.63, 54.57, 54.03, 50.58, 50.06, 49.88, 47.75, 47.18, 36.57, 35.58, 31.00, 29.49, 29.14, 27.89, 27.56, 27.20, 25.85, 24.70, 24.30, 22.27, 14.40. HR-MS *m*/*z* calcd for C_50_H_65_N_13_O_11_S_2_ 1087.4368, found [M + H]^+^ 1088.4383.

### Reduction stability experiment

Purified peptides (100 μg) was dissolved in 100 μL buffer (20 mM Tris–HCl, pH = 7.4, 150 mM NaCl, 1 mM CaCl_2_, 1 mM MgCl_2_, 0.1% Triton X-100). DTT was added to a final concentration of 3 mM. After 0, 0.5, 1, 2, 4 and 8 hours' incubation at 25 °C, the percent residual peptide was monitored by HPLC.

### Protease stability experiment

Purified peptides was dissolved in DMSO to a final concentration of 1 mM as solution A. α-Chymotrypsin was dissolved in PBS buffer (pH = 7.4, containing 2 mM CaCl_2_) to a final concentration of 0.5 ng μL^−1^ as solution B. 50 μL solution A and 1950 μL solution B were mixed. After 0, 0.5, 1, 2, 4, 8, 16 and 32 hours' incubation at 25 °C, the percent residual peptide was monitored by HPLC.

## Conflicts of interest

There are no conflicts to declare.

## Supplementary Material

RA-009-C8RA09761E-s001
