# Supplementary material for: pDobz/pDobb protected diaminodiacid as a novel building block for peptide disulfide-bond mimic synthesis
Source: RSC Adv. 2019 Feb 12;9(10):5438–44. doi: 10.1039/c8ra09761e (PMC9060753; doi:10.1039/c8ra09761e)
Supplement: RA-009-C8RA09761E-s001 [file RA-009-C8RA09761E-s001.pdf]

Electronic Supplementary Material (ESI) for RSC Advances.
This journal is © The Royal Society of Chemistry 2019

Supporting Information

pDobz/pDobb Protected Diaminodiacid as Novel Building Block for Peptide Disulfide-bond Mimics Synthesis

Chao Liu[a], Yan Zou[a], Honggang Hu[a], Yunyun Jiang[*b], Luping Qin[*ac]

Email: Shygm98@163.com (Yunyun Jiang); qinsmmu@126.com (Luping Qin)

a) *College of Pharmacy, Second Military Medical University, 325 Guohe Road, Shanghai, 200433, China*

b) *Department of Pharmacy, No.101 Hospital of PLA, Wuxi 214044, China*

c) *College of Pharmaceutical sciences, Zhejiang Chinese Medical University, Gaoke Road, Fuyang District, Hangzhou 311402, China*

1. Experimental Section

1.1 Synthesis of contryphan-Vn

To investigate reduction stability and protease stability, we synthesized the native model peptide contryphan-Vn shown in Scheme S1.

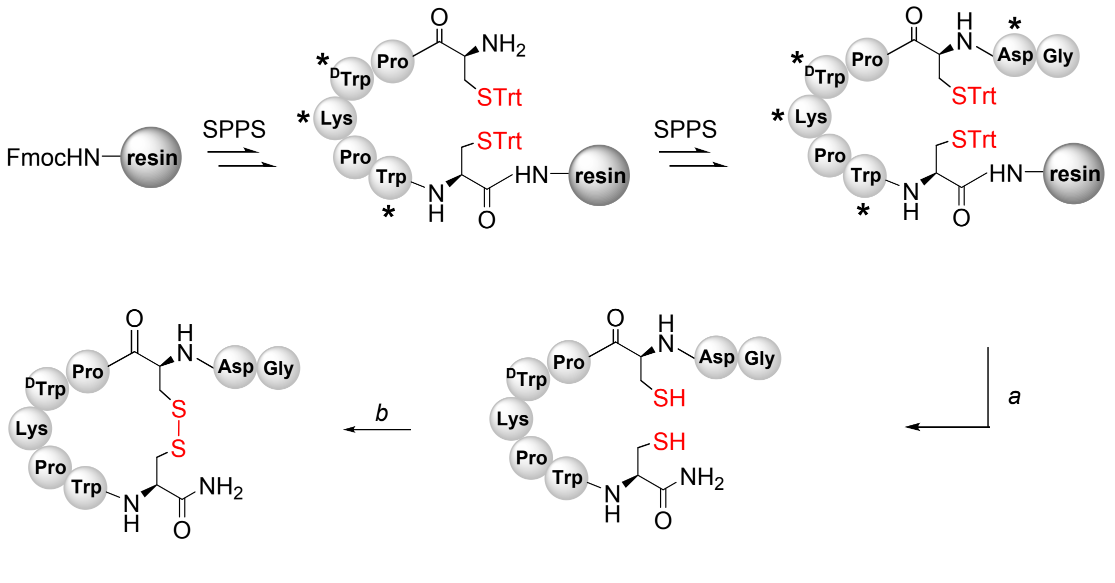

Scheme S1. Route of synthesis of contryphan-Vn; reagents and conditions: a) TFA/EDT/TIPs/water (95:2:2:1, *v/v/v/v*), rt, 2 h; b) Guanidine hydrochloride, sodium dihydrogen phosphate, PBS buffer (containing 10% DMSO), rt, 24 h; The resin-bound peptides were protected on side chains at asterisk sites. The following protecting groups for amino acid side chains were used: tert-butyl (tBu; for Asp), trityl (Trt; for Cys) and tert-butyloxycarbonyl (Boc; for Lys and Trp).

1.2 Characterization of contryphan-Vn

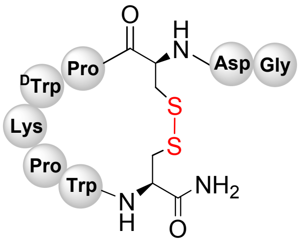

contryphan-Vn

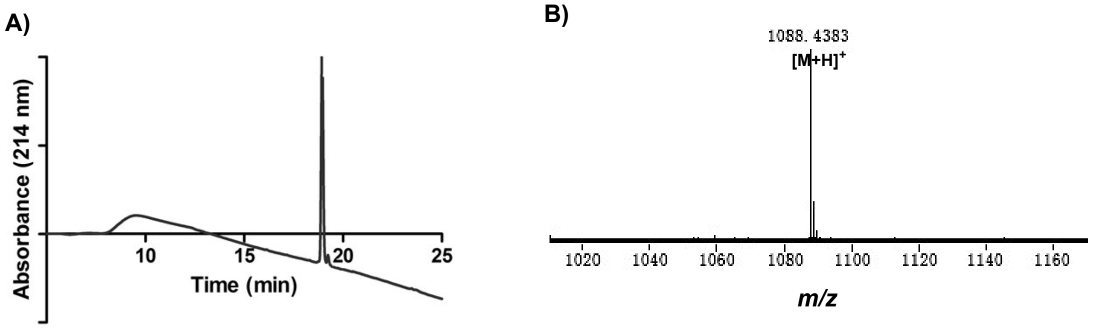

Figure S1. Contryphan-Vn was obtained as a white powder (46.8 mg, 43% yield according to initial resin load, 97.2% purity). a) HPLC trace of purified contryphan-Vn. Gradient: 90-0% of buffer B in 25 min with C18 column (5 μm, 4.6×250 mm). b) HR-MS spectrum of contryphan-Vn (calc. for $C_{50}H_{65}N_{13}O_{11}S_2$ 1087.4368, found $[M+H]^+$ 1088.4383).

2. HRMS data of small molecular compounds

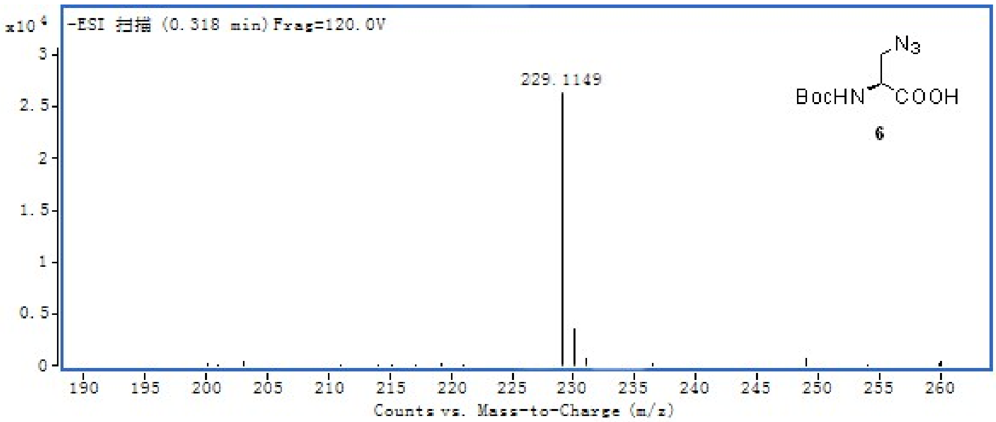

Figure S2. HR-MS spectrum of compound 6 (calc. for $C_8H_{14}N_4O_4$ 230.1015, found [M-H]$^-$ 229.1149).

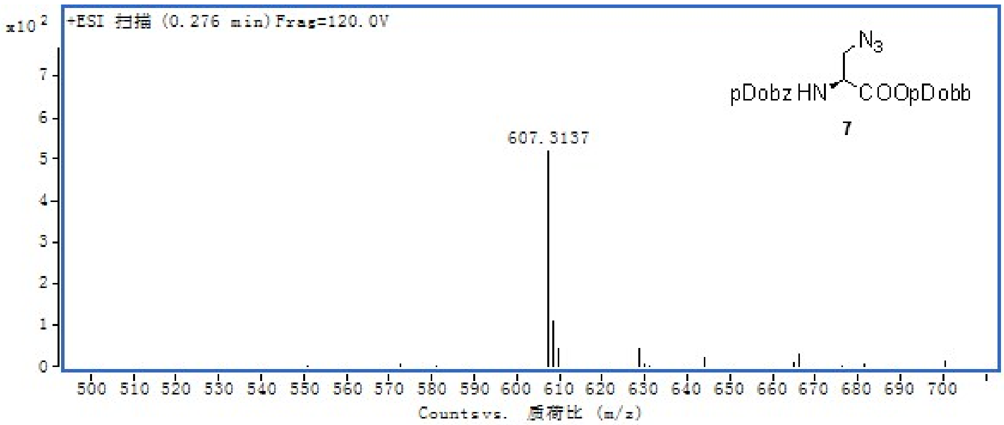

Figure S3. HR-MS spectrum of compound 7 (calc. for $C_{30}H_{40}B_2N_4O_8$ 606.3032, found [M+H]$^+$ 607.3137).

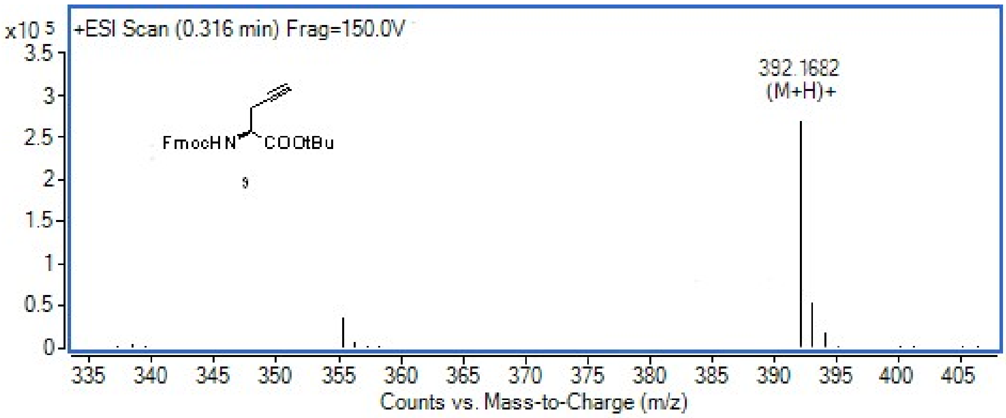

Figure S4. HR-MS spectrum of compound 9 (calc. for $C_{24}H_{25}NO_4$ 391.1784; found [M+H]$^+$ 392.1682).

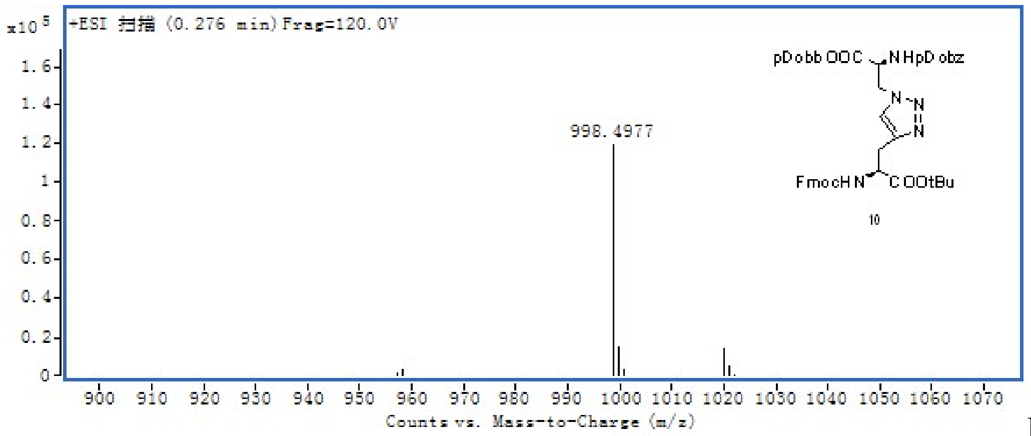

Figure S5. HR-MS spectrum of compound 10 (calc. for $C_{54}H_{65}B_2N_5O_{12}$ 997.4816; found $[M+H]^+$ 998.4977).

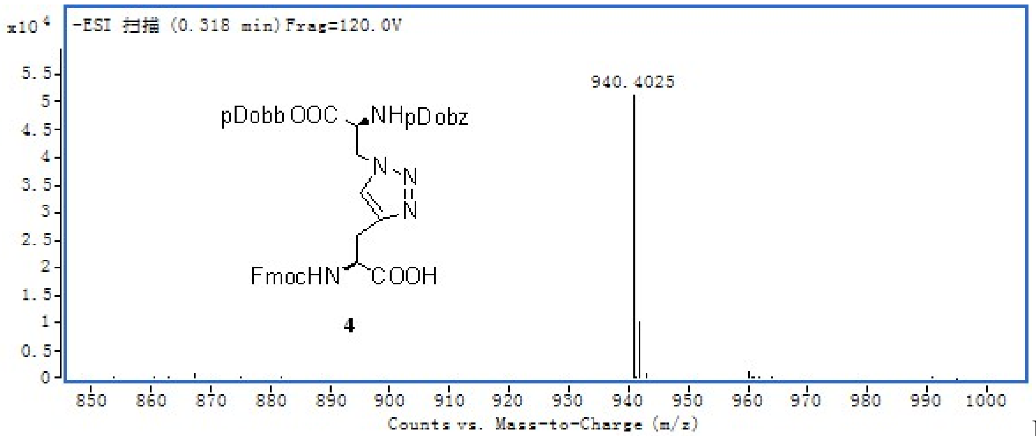

Figure S6. HR-MS spectrum of compound 4 (calc. for $C_{50}H_{57}B_2N_5O_{12}$ 941.4190; found $[M-H]^-$ 940.4025).

3. ^{1}H and ^{13}C NMR spectra of compounds

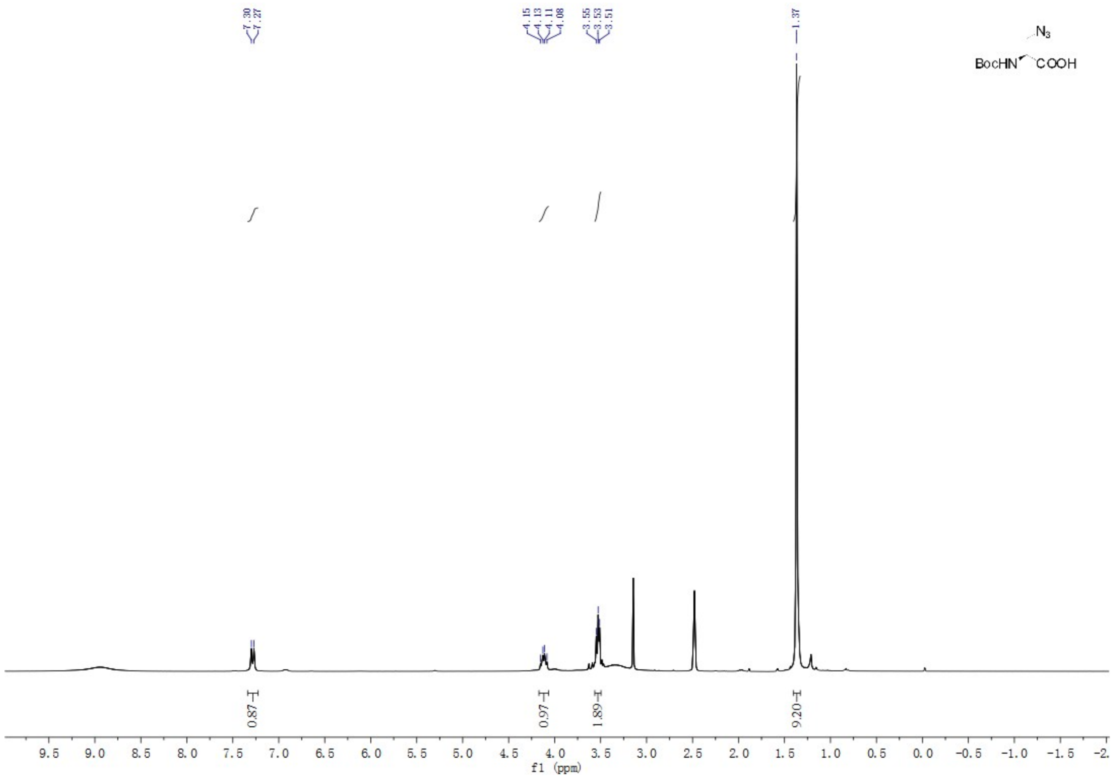

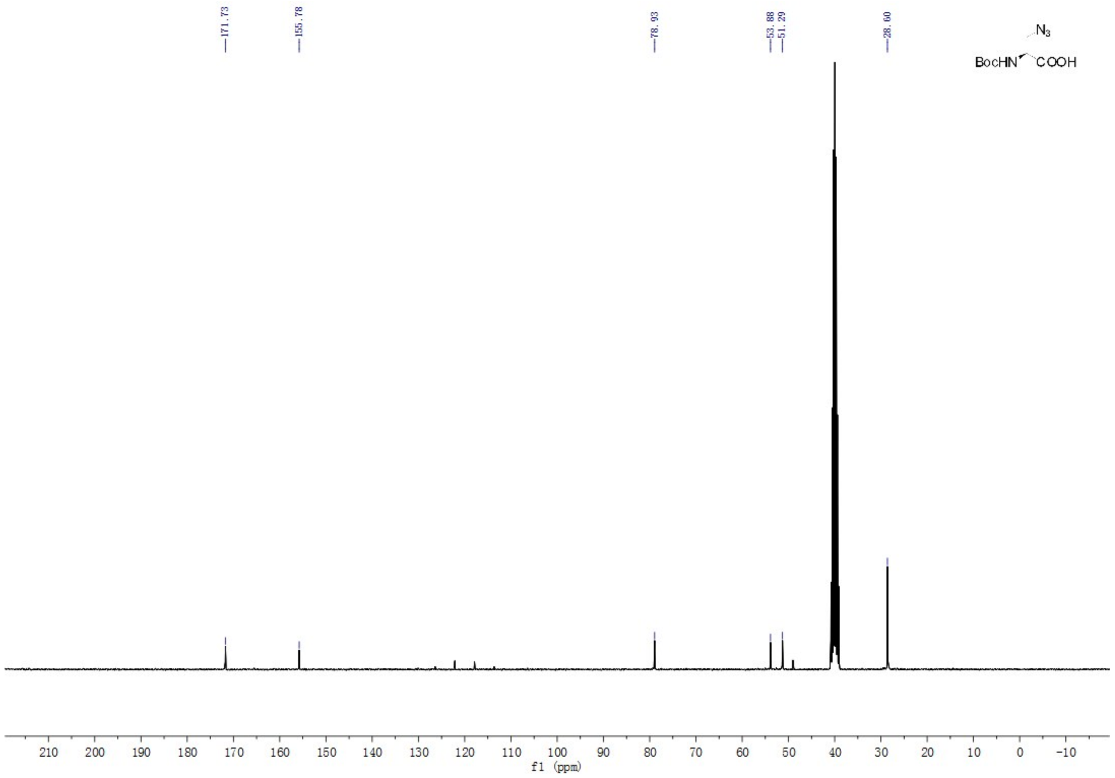

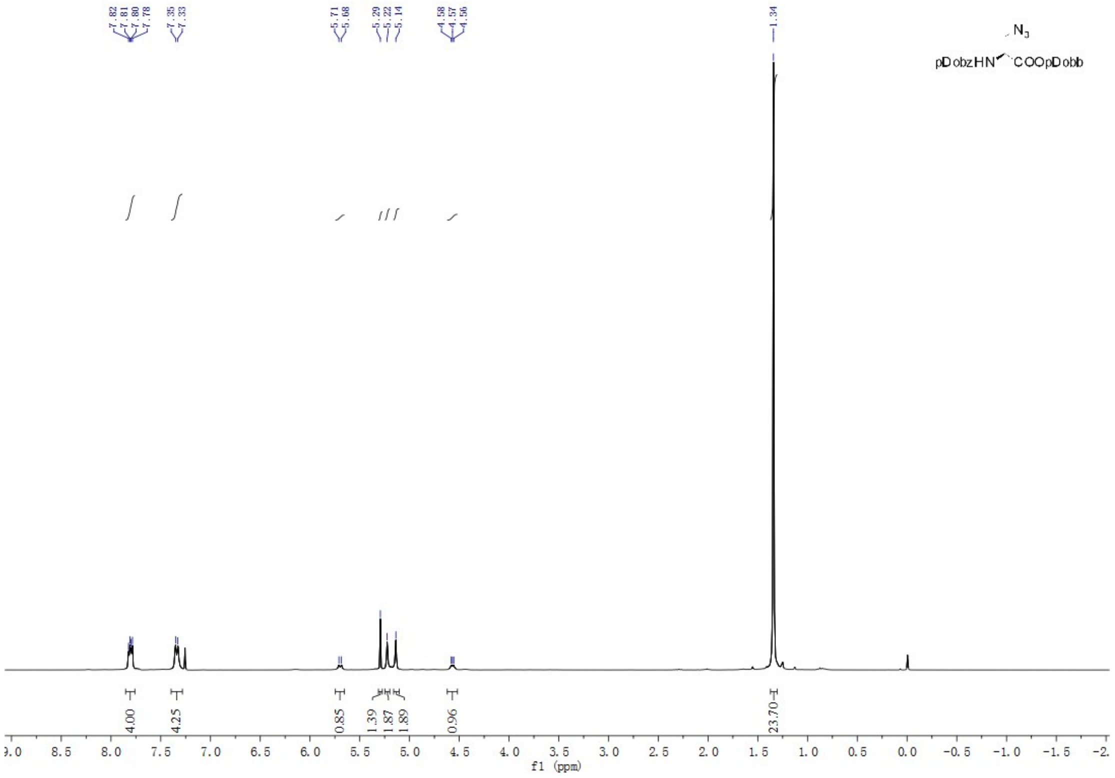
7.82
7.81
7.80
7.78
7.35
7.33
5.71
5.68
5.29
5.22
5.14
4.58
4.57
4.56
1.34
N3
pDobzHN COOpDobb
4.00
4.25
0.85
1.39
1.87
1.89
0.96
23.70
f1 (ppm)

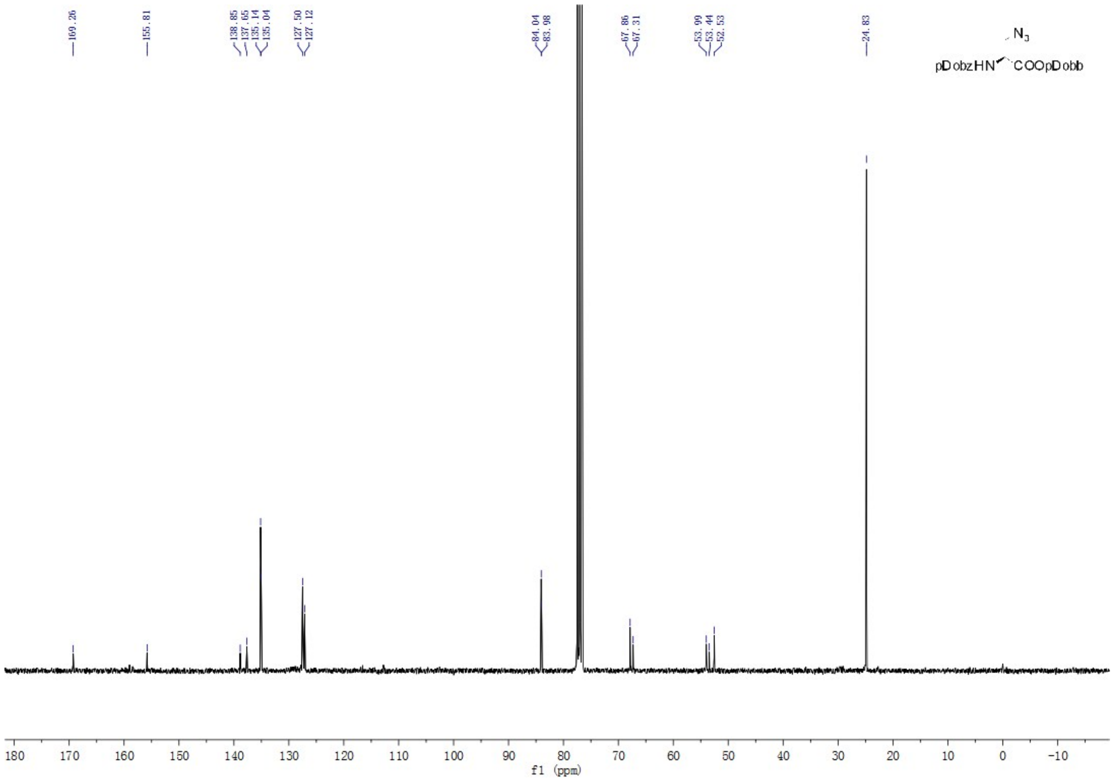
169.26
155.81
138.85
137.65
135.14
135.04
127.50
127.12
84.04
83.98
67.86
67.31
53.99
53.44
52.53
24.83
N3
pDobzHN COOpDobb
f1 (ppm)

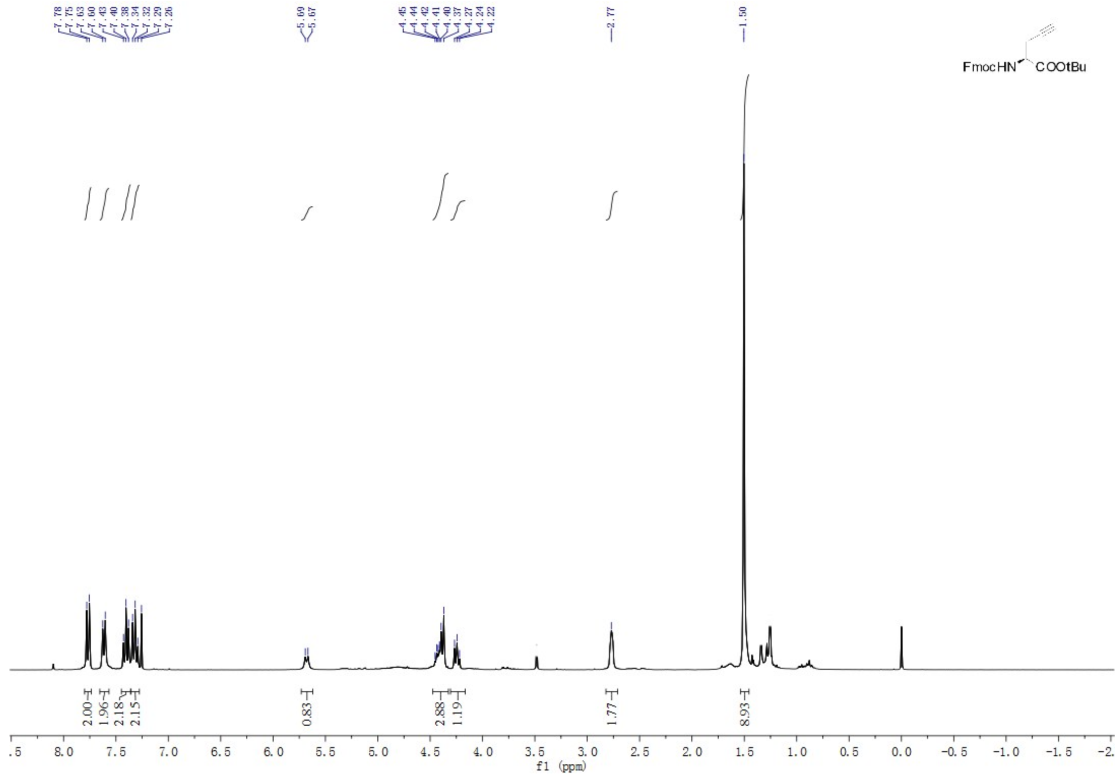
7.78 7.75 7.63 7.60 7.43 7.40 7.38 7.34 7.32 7.29 7.26
5.69 5.67
4.45 4.44 4.42 4.41 4.40 4.37 4.27 4.24 4.22
2.77
1.50
FmocHN COOtBu
2.00 1.96 2.18 2.15 0.83 2.88 1.19 1.77 8.93
f1 (ppm)

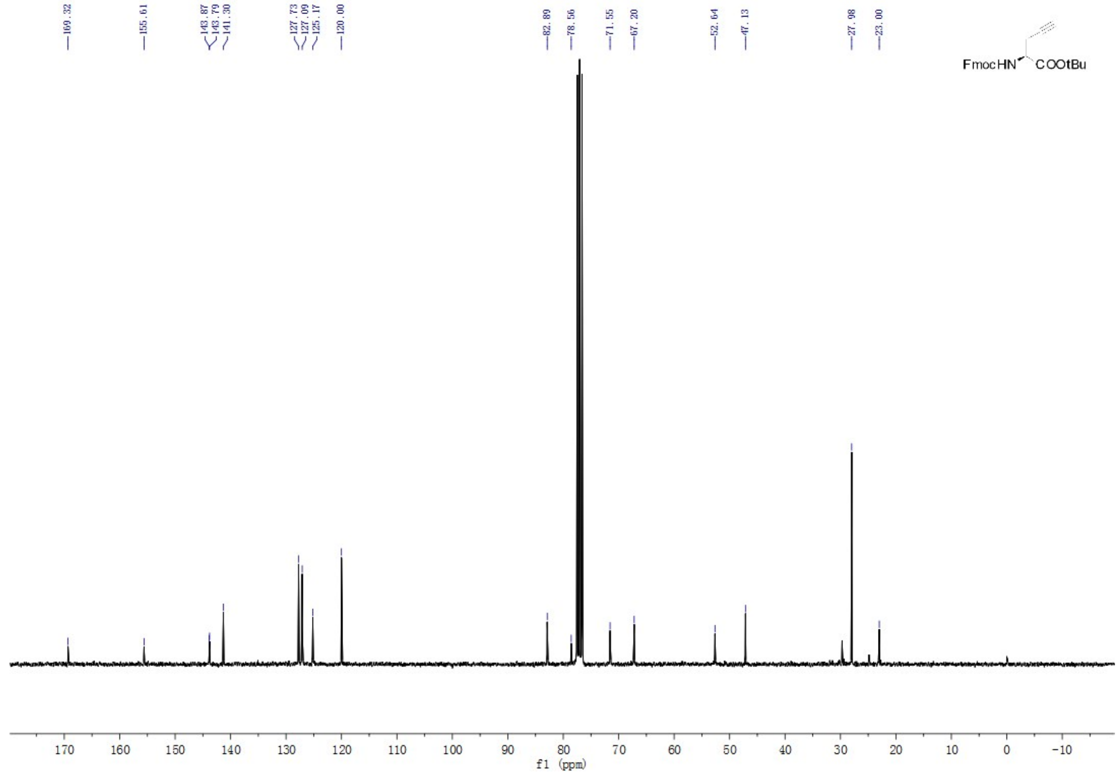
169.32
155.61
143.87 143.79 141.30
127.73 127.09 125.17 120.00
82.89 78.56 71.55 67.20
52.64 47.13
27.98 23.00
FmocHN COOtBu
f1 (ppm)

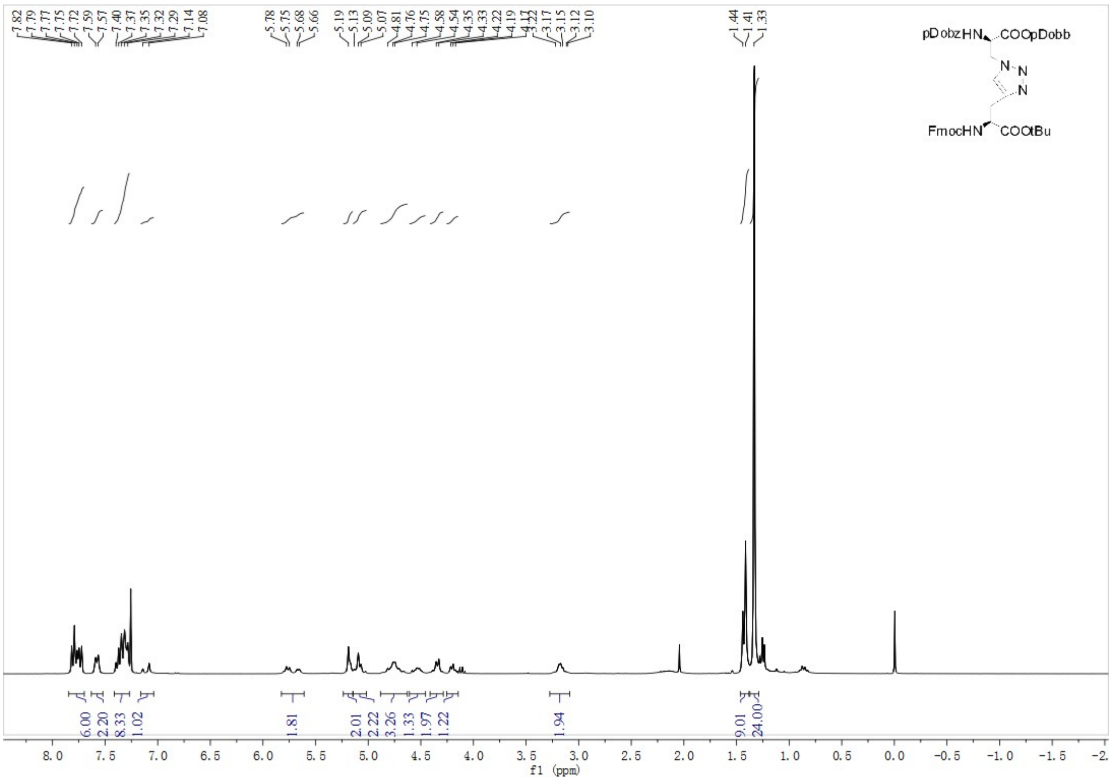

7.82
7.79
7.77
7.75
7.72
7.59
7.57
7.40
7.37
7.35
7.32
7.29
7.14
7.08
5.78
5.75
5.68
5.66
5.19
5.13
5.09
5.07
4.81
4.76
4.75
4.58
4.54
4.35
4.33
4.22
4.19
4.13
3.22
3.17
3.15
3.12
3.10
1.44
1.41
1.33
pDobzHN COOpDobb
N N N
FmocHN COOtBu
6.00
2.20
8.33
1.02
1.81
2.01
2.22
3.26
1.33
1.97
1.22
1.94
9.01
24.00
f1 (ppm)

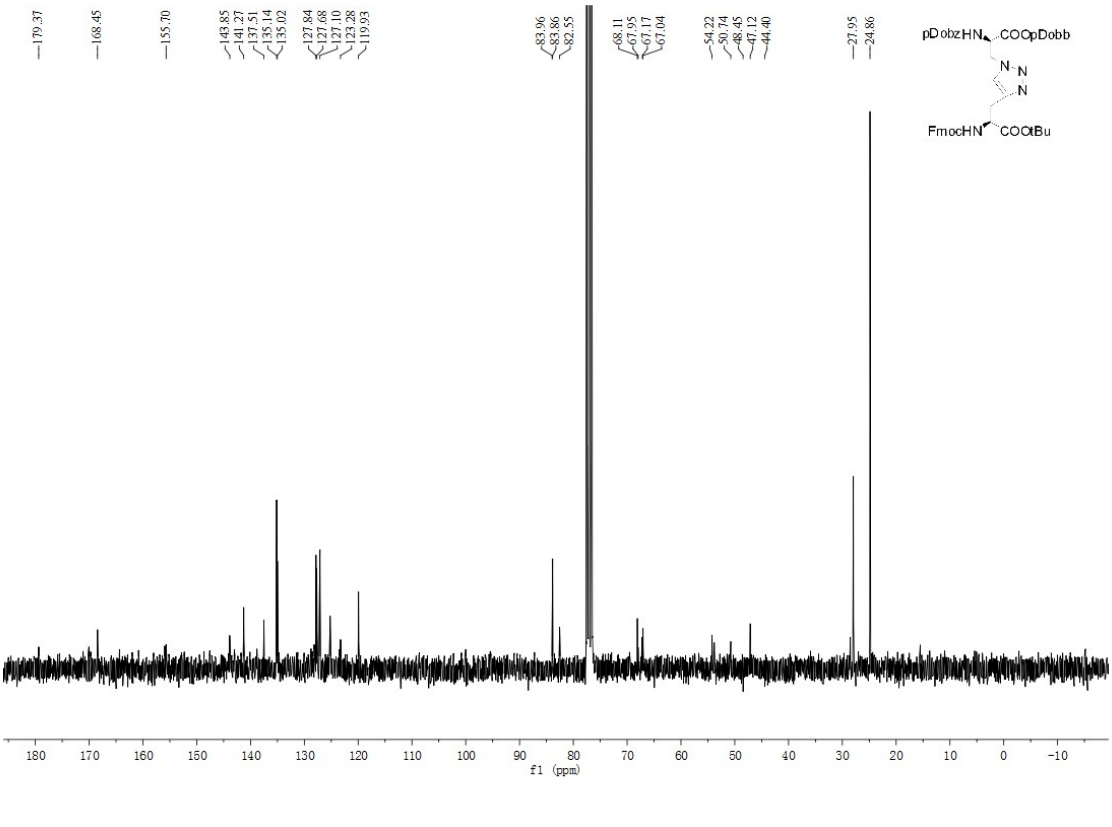

179.37
168.45
155.70
143.85
141.27
137.51
135.14
135.02
127.84
127.68
127.10
123.28
119.93
83.96
83.86
82.55
68.11
67.95
67.17
67.04
54.22
50.74
48.45
47.12
44.40
27.95
24.86
pDobzHN COOpDobb
N N N
FmocHN COOtBu
f1 (ppm)

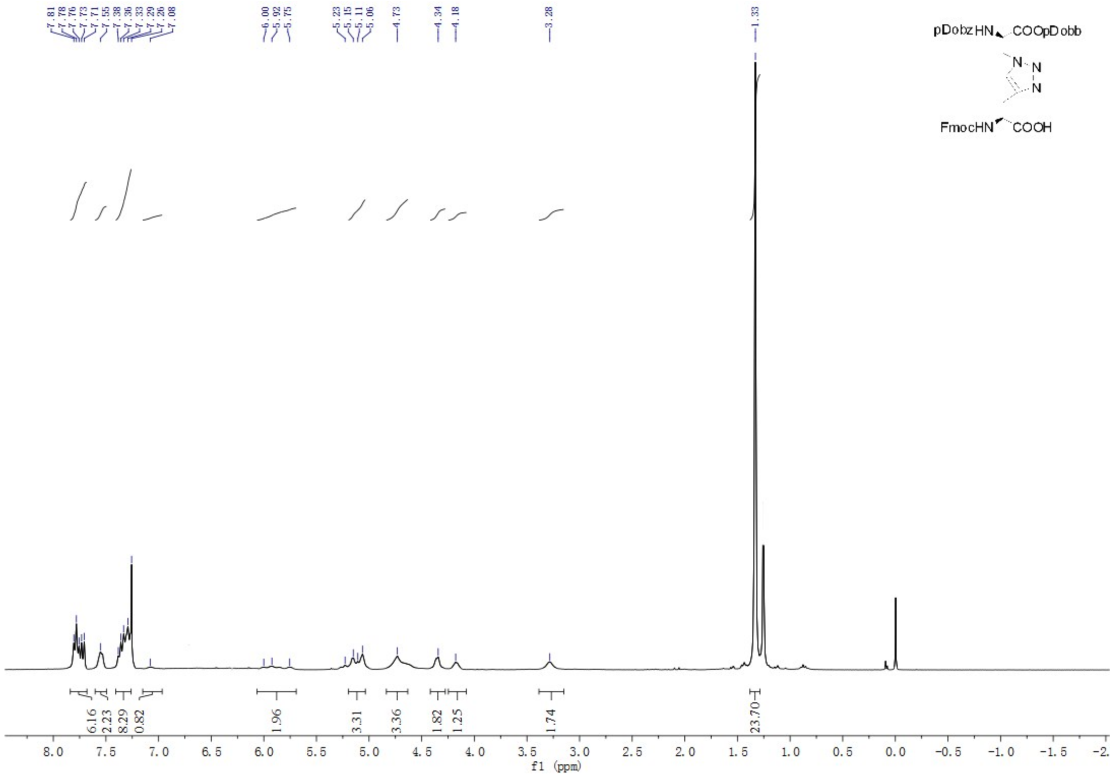
pDobzHN COOpDobb
N N N
FmocHN COOH
f1 (ppm)

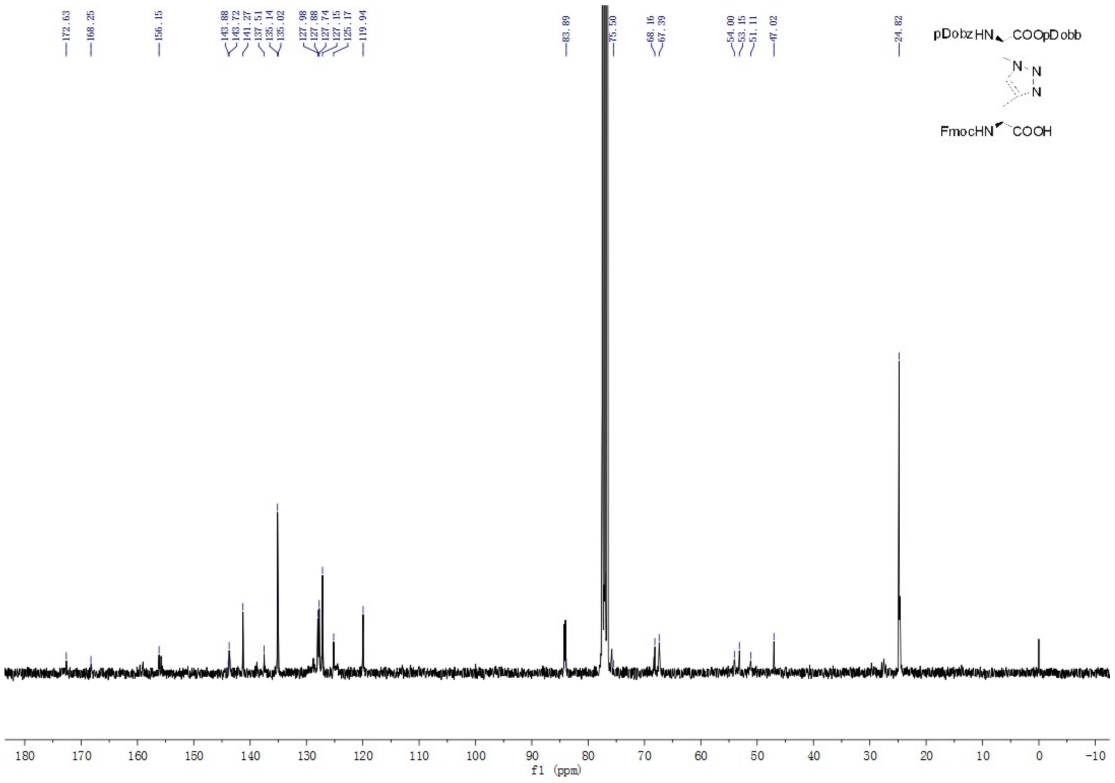
pDobzHN COOpDobb
N N N
FmocHN COOH
f1 (ppm)

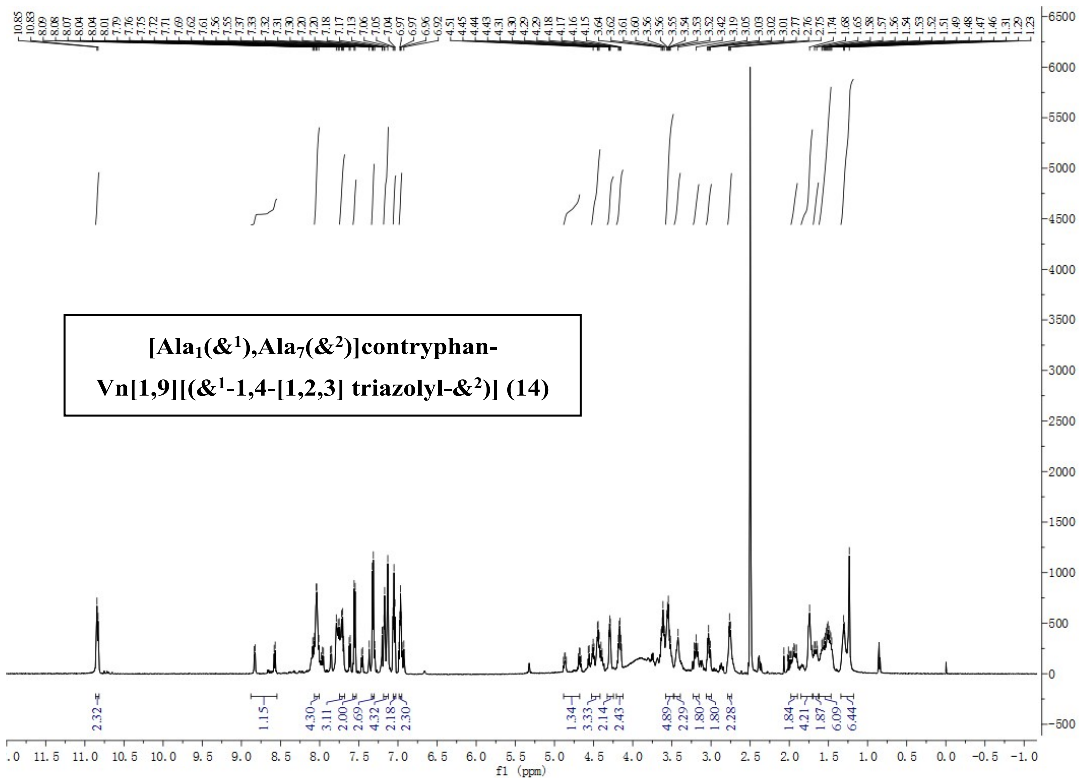
[Ala1(&1),Ala7(&2)]contryphan-
Vn[1,9][(&1-1,4-[1,2,3] triazolyl-&2)] (14)
f1 (ppm)

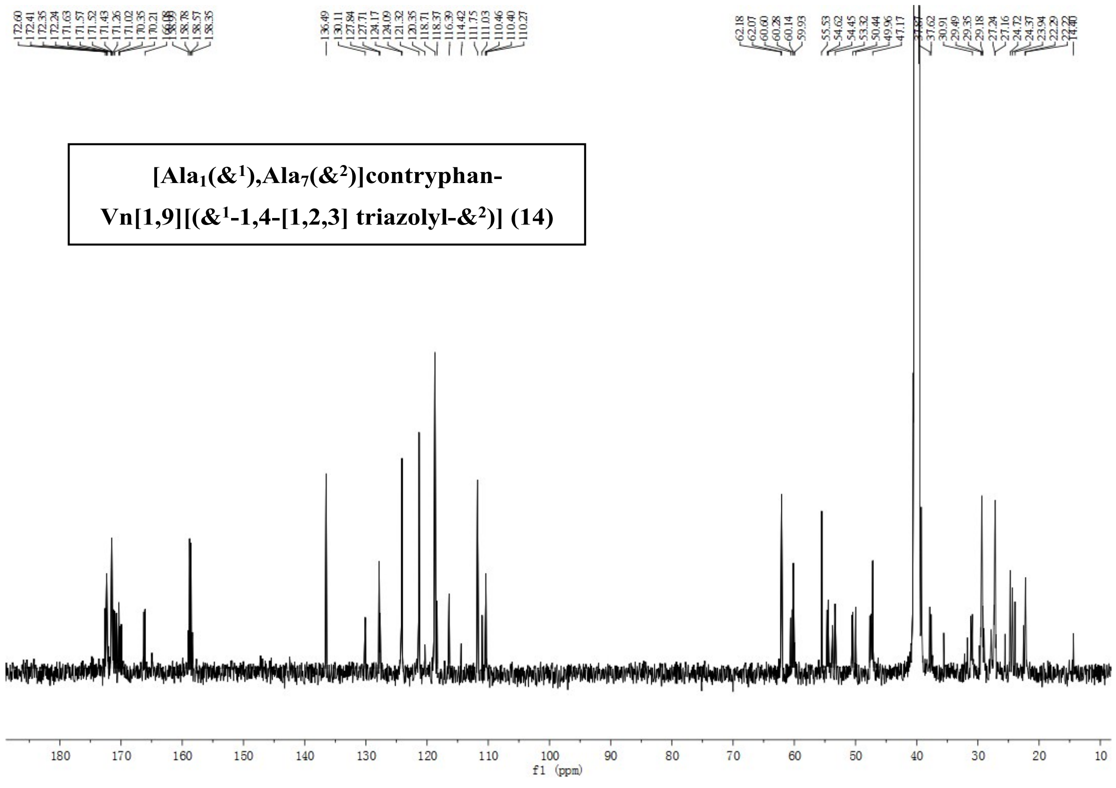
[Ala1(&1),Ala7(&2)]contryphan-
Vn[1,9][(&1-1,4-[1,2,3] triazolyl-&2)] (14)
f1 (ppm)

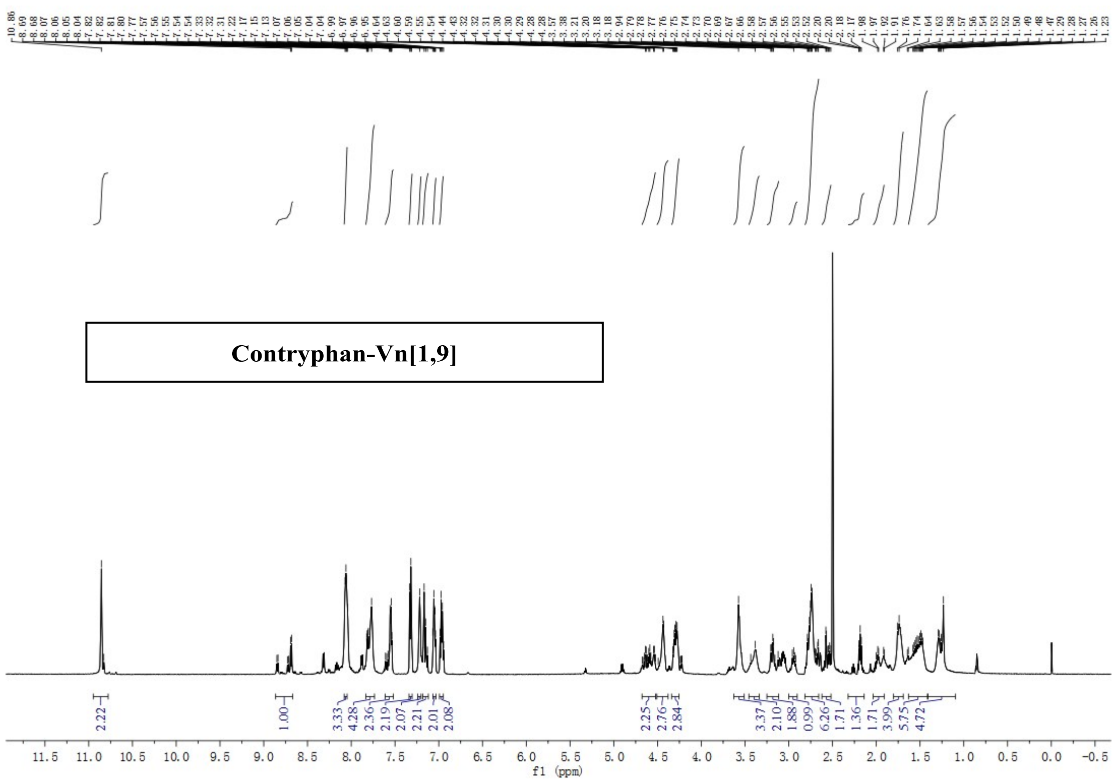
Contryphan-Vn[1,9]

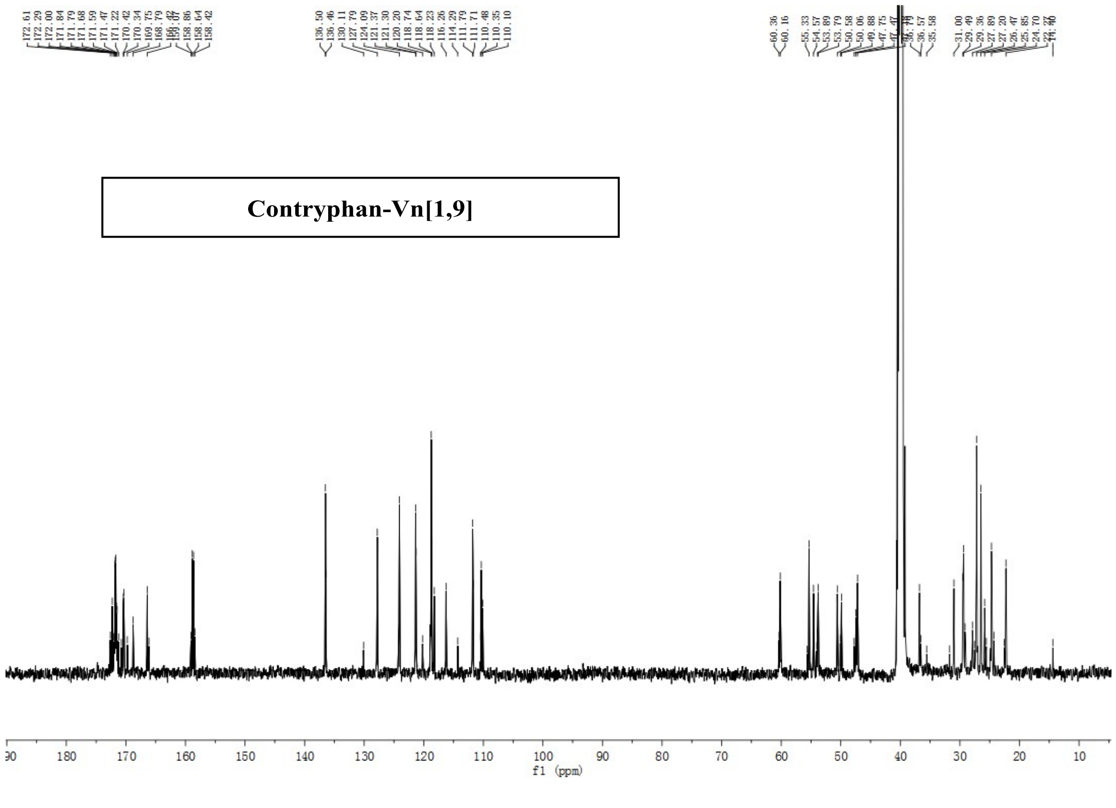
Contryphan-Vn[1,9]
